# Rounding creatinine, cystatin C or both: impact on discordance group assignment and GFR-estimating equation performance

**DOI:** 10.1093/ndt/gfad224

**Published:** 2023-10-18

**Authors:** Dion Groothof, Naser B N Shehab, Adrian Post, Reinold O B Gans, Stephan J L Bakker, Nicole S Erler

**Affiliations:** Department of Internal Medicine, Division of Nephrology, University Medical Center Groningen, University of Groningen, Groningen, The Netherlands; Department of Internal Medicine, Division of Nephrology, University Medical Center Groningen, University of Groningen, Groningen, The Netherlands; Department of Internal Medicine, Division of Nephrology, University Medical Center Groningen, University of Groningen, Groningen, The Netherlands; Department of Internal Medicine, Division of Nephrology, University Medical Center Groningen, University of Groningen, Groningen, The Netherlands; Department of Internal Medicine, Division of Nephrology, University Medical Center Groningen, University of Groningen, Groningen, The Netherlands; Department of Biostatistics, Erasmus Medical Center, Erasmus University Rotterdam, Rotterdam, The Netherlands; Department of Epidemiology, Erasmus Medical Center, Erasmus University Rotterdam, Rotterdam, The Netherlands

To the Editor,

Accurate estimation of glomerular filtration rate (GFR) is integral to routine medical care and clinical decision-making. Current guidelines endorse using serum creatinine as the initial test and an estimating equation to derive GFR [1]. If this estimated GFR (eGFR) is considered less accurate (e.g. due to lower-than-average muscle mass [2]), confirmatory testing with muscle mass–independent cystatin C is recommended [1]. However, evidence relating to the performance of cystatin C as GFR marker in real-world settings is lacking and is confined to research settings [3]. A recent study addressing this knowledge gap found that eGFR based on both creatinine and cystatin C (eGFRcr-cys) outperforms GFR derived from creatinine (eGFRcr) or cystatin C (eGFRcys) in a real-world setting, particularly when eGFRcr and eGFRcys are discordant [4]. Importantly, this and another recent study [5] presented cystatin C values as integers, a less informative format than the typically used real numbers [3, 6–8], especially when rounding from one to zero decimals. However, the impact of rounding levels of kidney function markers on the performance of GFR-estimating equations has never been quantified. This is a crucial area to explore, as rounding may influence the performance of GFR-estimating equations, potentially compromising clinical decision-making and invalidating conclusions from studies.

To explore this area, we followed previous methodology for developing GFR-estimating equations [3, 4] and quantified the impact of rounding creatinine, cystatin C or both on subject assignment to discordance groups [4] and the performance of GFR-estimating equations [3, 4] using data from 1184 adults referred for iothalamate clearance at the University Medical Center Groningen, Groningen, The Netherlands. GFR was measured by continuous low-dose infusion of the radiolabelled tracer ^125^I-iothalamate, as described elsewhere [9]. After a 2-h stabilization period, urinary clearances of ^125^I-iothalamate were calculated and corrected for possible voiding errors (e.g. incomplete bladder emptying and dead space) by multiplying by the ratio of the plasma and urinary clearance of ^131^I-hippuran. The day-to-day GFR variability is 2.5% [9]. We used the new creatinine-based Chronic Kidney Disease Epidemiology Collaboration (CKD-EPI) equations from 2021, which omit race, to compute eGFRcr and eGFRcr-cys. We used the cystatin C–based CKD-EPI equation from 2012 to compute eGFRcys, as it was not updated because it does not include race [3]. We assessed the performance of GFR-estimating equations under the various rounding scenarios by computing the bias (systematic error) as the median difference between measured GFR and eGFR, the interquartile range of the difference between measured GFR and eGFR as a measure of imprecision, and the percentage of estimates less than 30% different from the measured GFR (referred to as the P30). Confidence intervals around these performance measures were obtained via bootstrapping, stratified by discordance groups to maintain the proportion of subjects within discordance groups. Statistical analyses were performed with R version 4.3.1 (Vienna, Austria).

The mean (standard deviation) age, measured GFR, eGFRcr, eGFRcys and eGFRcr-cys of the total sample (49.8% male) were 56.1 (11.2) years and 87.3 (29.7), 74.3 (22.0), 73.1 (26.0) and 75.8 (24.6) mL/min/1.73 m^2^, respectively. Median (25th; 75th percentile) serum creatinine and cystatin C were 1.03 (0.85; 1.30) mg/dL and 1.05 (0.85; 1.28) mg/L, respectively. Using two decimals for both markers, 229 (19.3%) subjects were assigned to the eGFRcys < eGFRcr discordance group. Rounding creatinine, cystatin C or both markers to one decimal resulted in respective assignment of 235 (19.8%), 229 (19.3%) and 232 (19.6%) subjects to this discordance group, while rounding to zero decimals resulted in respective assignment of 306 (25.8%), 377 (31.8%) and 87 (7.3%) subjects to this discordance group, consistent with the larger information loss in this rounding scenario. While 799 (67.5%) subjects were assigned to the eGFRcys ≈ eGFRcr concordance group when using two decimals for both markers, rounding of creatinine, cystatin C or both markers to one decimal resulted in respective assignment of 782 (66.0%), 797 (67.3%) and 783 (66.1%) subjects, and rounding to zero decimals resulted in respective assignment of 509 (43.0%), 550 (46.5%) and 1048 (88.5%) subjects to this group. For the eGFRcys > eGFRcr discordance group, 156 (13.2%) subjects were assigned using two decimals for both markers. Rounding creatinine, cystatin C or both markers to one decimal resulted in respective assignment of 167 (14.1%), 158 (13.3%) and 169 (14.3%) subjects and rounding to zero decimals resulted in respective assignment of 369 (31.2%), 257 (21.7%) and 49 (4.1%) subjects, respectively, to this group.

Rounding either marker or both simultaneously from two to one decimal appeared to have negligible effect on the performance of all estimating equations across discordance groups, while rounding from one to zero decimals substantially affected performance (Fig. 1A–C). Therefore, we focused on the latter rounding scenario for further result description. In the eGFRcys < eGFRcr discordance group, rounding creatinine from one to zero decimals significantly shifted the median biases (95% CI) of eGFRcr and eGFRcr-cys from-1.25 (4.25; 1.40) and –11.34 (–13.39; –8.66) to 8.13 (6.69; 9.11) and –5.54 (–6.72; –4.42) mL/min/1.73 m^2^, respectively. Moreover, rounding of cystatin C from one to zero decimals significantly shifted the median biases of eGFRcr, eGFRcys and eGFRcr-cys from –0.73 (–3.63; 1.48), –20.08 (23.00; 16.65) and 11.25 (13.66; –8.63) to 10.22 (12.28; 8.10), 33.47 (35.76; –31.43) and –22.60 (24.20; 19.79) mL/min/1.73 m^2^, respectively. Finally, rounding both from one to zero decimals significantly shifted the median biases of eGFRcr from –0.95 (–3.72; 1.50) to 12.45 (8.48; 16.01) mL/min/1.73 m^2^ (Fig. 1A). In the eGFRcys ≈ eGFRcr concordance group, rounding affected performance measures only minimally (Fig. 1B). In the eGFRcys > eGFRcr discordance group, eGFRcr was the estimating equation affected most by rounding. Rounding creatinine from one to zero decimals significantly increased the median bias of eGFRcr and eGFRcr-cys from –20.00 (21.49; –17.66) and –6.30 (–9.09; –3.54) to 32.80 (35.54; –31.74) and –15.13 (–18.02; –13.44) mL/min/1.73 m^2^, respectively. Furthermore, rounding both markers from one to zero decimals likewise increased the median bias of eGFRcr from –19.91 (21.24; –16.60) to 32.99 (37.18; 29.43) mL/min/1.73 m^2^ (Fig. 1C).

**Figure 1:**
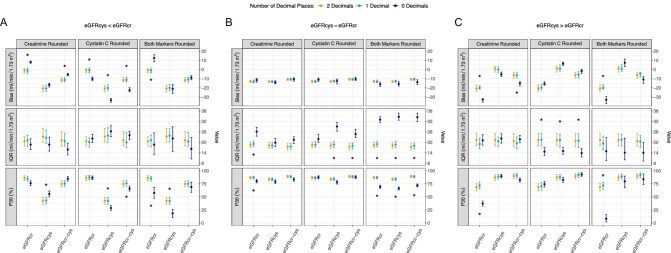
Comparison of the performance of GFR-estimating equations stratified by discordance groups under various rounding conditions for levels of serum creatinine and/or cystatin C. A subject was assigned to the eGFRcys < eGFRcr discordance group when eGFRcys was more than 20% lower than eGFRcr (Panel A); the eGFRcys ≈ eGFRcr concordance group when eGFRcys was within 20% of eGFRcr (Panel B); and the eGFRcys > eGFRcr discordance group when eGFRcys was more than 20% higher than eGFRcr (Panel C). Each row of plots shows a different performance measure, whereas the columns contain the different scenarios of rounding serum creatinine (in mg/dL), serum cystatin C (in mg/L) or both markers simultaneously. The median bias was defined as the mid-point of the distribution of the difference between mGFR and eGFR (i.e. mGFR – eGFR). Therefore, a positive (negative) bias implies underestimation (overestimation) of the mGFR. The IQR was defined as the 75th percentile of the bias subtracted from the 25th percentile of the bias and is considered as a measure of imprecision. The P30 was defined as the percentage of subjects with an eGFR within 30% of the mGFR. Shown are the performance measures and corresponding 95% bootstrap confidence intervals. Differences in performance measures when rounding to one versus zero decimal places with non-overlapping 95% confidence intervals are indicated with an asterisk. cr, creatinine; cys, cystatin C; IQR, interquartile range; mGFR, measured glomerular filtration rate.

Rounding the first decimal of creatinine and cystatin C values to zero decimals appears to substantially affect both the assignment of subjects to discordance groups and the resulting performance of estimating equations to levels that may severely compromise decision-making and invalidate study conclusions. This issue is also of importance in the study of selective glomerular hypofiltration syndromes, which uses the ratio of eGFRcys to eGFRcr as criterion [10]. Using integers instead of real numbers for creatinine or cystatin C may inadvertently introduce bias into these metrics, potentially misdirecting clinical and research outcomes.

With two US national kidney disease organizations recently advocating for more routine use of cystatin C [11], clinicians will increasingly face discordant values between eGFRcr and eGFRcys. Discordant values, currently an active area of research [4, 5, 8, 10, 12], imply that eGFRcr, eGFRcys or both differ from measured GFR. Our work sheds light on how using integers instead of real numbers for creatinine and cystatin C levels affects GFR estimation, highlighting one contributing factor to discordant eGFR values. However, several other factors can also produce discordant values.

Measurement errors, which can arise during three distinct phases in the laboratory testing process, as detailed elsewhere [13], can contribute to the production of discordant values. The pre-analytical phase of the laboratory testing process entails challenges related to patient assessment, specimen collection, transport and laboratory receipt. The analytical phase encompasses all steps from specimen preparation to test result verification and, despite a decline in errors recently, still faces challenges like improper specimen processing, instrument-related errors and assay-related errors. Assay-related errors include sensitivity, specificity, within-run and between-run variability, and interfering substances—typically being well-documented quantities in assay manuals. The post-analytical phase focusses on effective communication and interpretation of results. Altogether, flaws in each of these phases can accumulate errors, which can substantially affect eGFR accuracy.

The assumptions underlying different GFR-estimating equations can also influence eGFR accuracy, depending on the usage context. Inclusion of age and sex as variables in currently recommended GFR-estimating equations [11] partially accounts for variability in creatinine and cystatin C levels unrelated to GFR, known as ‘non-GFR determinants’ [3]. However, this inherently holds the assumption that non-GFR determinants are consistent among individuals of identical age and sex, which leaves eGFR subject to residual confounding if this assumption is not met [2]. Moreover, there may be (unidentified) non-GFR determinants unrelated to age and sex that affect eGFR [1].

In summary, our findings underscore the necessity of adhering to a logical set of rules for appropriate rounding of biological measurements, a process that involves gathering the expanded uncertainty of a particular assay and identifying the number of significant decimals [14]. For serum creatinine (in mg/dL) and cystatin C (in mg/L), this means using real numbers with two decimal places as per typical measurement imprecision of assays. Adherence to these rules will maximize accuracy in both clinical practice and research.

## Data Availability

The data underlying this article and R code to implement the analyses will be shared on reasonable request to the corresponding author.
